# Clinical features and surgical outcomes of primary canaliculitis with concretions

**DOI:** 10.1097/MD.0000000000006188

**Published:** 2017-03-03

**Authors:** Shengjin Xiang, Bin Lin, Qintuo Pan, Meiqin Zheng, Xiaoyi Qin, Youpei Wang, Zongduan Zhang

**Affiliations:** a Eye Hospital of Wenzhou Medical University, Wenzhou; b Department of Ophthalmology, the Second Hospital of Yinzhou, Ningbo, China.

**Keywords:** canalicular concretions, canaliculitis, canaliculotomy, curettage, histopathology, microbiology

## Abstract

The purpose of our study is to analyze the clinical, ultrasonic, microbiologic, and histopathologic characteristics, management, and outcomes in a series of primary canaliculitis with concretions patients who underwent canaliculotomy with curettage.

Thirty-six patients were reviewed for age, sex, location and laterality, duration of symptoms, clinical symptoms, ultrasonic signs, result of microbiologic culture and histopathologic examination, treatment, and outcomes. Main outcomes were the clinical, ultrasonic, and microbiological characteristics of the canalicular concretions; the histopathologic profiles; and the treatment effect.

Thirty-six patients were identified with concretions in all 37 cases of the patients with canaliculitis. There were 30 (83.3%) female patients with a mean age of 54.2 years. Twenty-eight (77.8%) patients were misdiagnosed or delayed diagnosed, and the mean duration was 17.1 months. The common most clinical presentations were discharge (100%), epiphora (66.7%), erythema (52.8%), and swelling (47.2%), and concretions were found in 31 of 37 patients by typical clinical manifestations and in 5 of 6 patients by ultrasonic. *Actinomyces* was found in 8 of 13 histopathologic specimens, and microbiological cultures were positive in 13 of 24 patients. All patients underwent canaliculotomy with curettage to completely remove all concretions and contents; 35 of 36 patients’ symptoms improved and 1 recurred after treatment at a median of 21.7 months follow-up according to the telephonic questionnaires.

Canalicular concretions play an important role in primary canaliculitis. Canaliculotomy with curettage is a standard therapy with canalicular concretions, and the surgical removal of all possible concretions is essential for cure.

## Abstract 2

Supplemental Digital Content is available in the text

## 1 Introduction

Canalicular concretions are a common finding in patients with primary canaliculitis, and its existence must lead to canaliculitis.^[1–8]^ However, canaliculitis is often accompanied by canalicular concretions, but it is often overlooked or misdiagnosed and treated insufficiently for limited morbidity.^[2,9]^ Importantly, the presentation of concretions was associated with a higher recurrent rate and failure for conservative therapy in patient of canaliculitis.^[3,8,10,11]^ Therefore, the understanding of the clinical characteristics and signs, treatment methods of primary canaliculitis with concretions, and a comprehensive literature review about canaliculitis with concretions may contribute to avoiding a misdiagnosis and reducing the recurrence of canaliculitis.

There are many studies that summarized the clinical characteristics and treatment of primary canaliculitis, but there are few literature that investigated primary canaliculitis with concretions absolutely. Berlin et al and Repp et al^[2,6]^ compared the characteristics of lacrimal-sac dacryoliths and canalicular concretions; meanwhile, canalicular concretions were noted in all primary canaliculitis patients in a few study,^[2–7]^ so the clinical symptoms and signs of canaliculitis also represent the clinical features of canaliculitis with concretions, but all of the those findings were based on a small sample of cases. To better characterize canalicular concretions in our current study, we retrospectively investigated a large case series of patients with canalicular concretions, and analyzed the clinical presentation, diagnosis, and treatment of primary canaliculitis with concretions. Meanwhile, a comprehensive literature review was also conducted to help establish an up-to-date summary of canalicular concretions.

## 2 Methods

We retrospectively reviewed the medical records with canaliculitis patients who were examined in the Eye Hospital of Wenzhou Medical University from January 2008 to September 2016. This study was approved by the ethics committee of the Eye Hospital of Wenzhou Medical University, and all subjects or their legal guardians gave written informed consent to participate. The following data were obtained from the patients’ clinical records: age, sex, presenting symptom, duration between onset of symptoms and diagnosis, involved side and location, results of the microbiologic culture, pathology, ultrasonic image, treatment and outcomes. Most patients who were diagnosed with canalicular concretions were based on the presence of discharge at morning, epiphora, eyelid erythema, canalicular swelling, expression of discharge or concretions from the punctum, and the experience of the clinician, and few patients were diagnosed by ultrasonic with 20-MHZ. All patients were identified by screening the clinical and surgical management records and/or pathology laboratory database. No concretions and secondary canaliculitis (means lacrimal smartplug-related canaliculitis) were excluded in this study.

All patients underwent surgical intervention including canaliculotomy with curettage, which was performed by cutting the punctum along the posterior wall with small, straight scissors, and then repressing the canaliculus and removing the concretions completely. If not, a horizontal incision approximately 6 to 8 mm in length should be made through the eyelid margin to open the canaliculus, and then remove the concretions or mucoid debris and granulation tissue completely with a 2-mm chalazion curette, irrigating the end with antibiotic solution. The concretions were then collected and sent to the ophthalmic laboratory for further microbiologic culture and histopathologic diagnosis. After surgery, all patients were treated with antibiotic eye drops (levofloxacin and/or sulfacetamide) 4 times daily and then refined according to culture results and sensitivities for 3 weeks.

To assess the patients’ treatment outcomes regarding recurrence rates and symptoms of discharge and epiphora, we surveyed subjects using a telephonic questionnaire. The survey items included queries about chronic symptoms of mucopurulent discharge, epiphora, and erythema. The outcomes included complete resolution, partial resolution, and recurrence of symptoms and/or signs.

The PubMed database was searched to identify articles up to June 2016 using the following terms: canaliculitis, canalicular concretions, canaliculitis, and concretions; and canalicular and stone. Results were limited to English articles in humans. The reference sections of these articles were then reviewed to identify further articles. Table 1 summarizes the results of the literature review about canaliculitis in all patients with canalicular concretions in English.

**Table 1:**
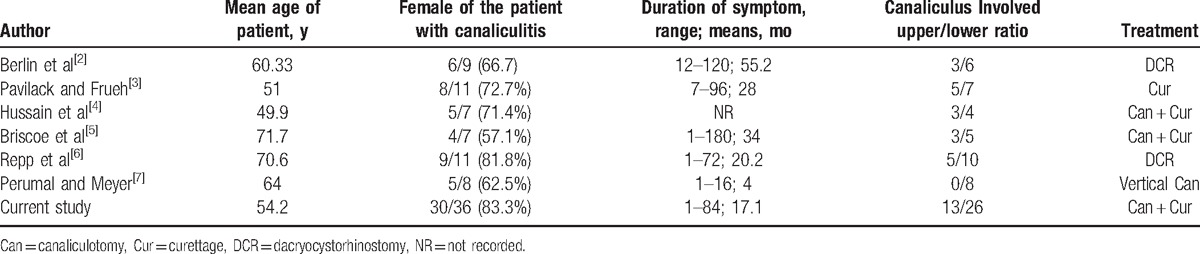
Literature review of canaliculitis in all patients with canalicular concretions.

## 3 Results

**Figure 1:**
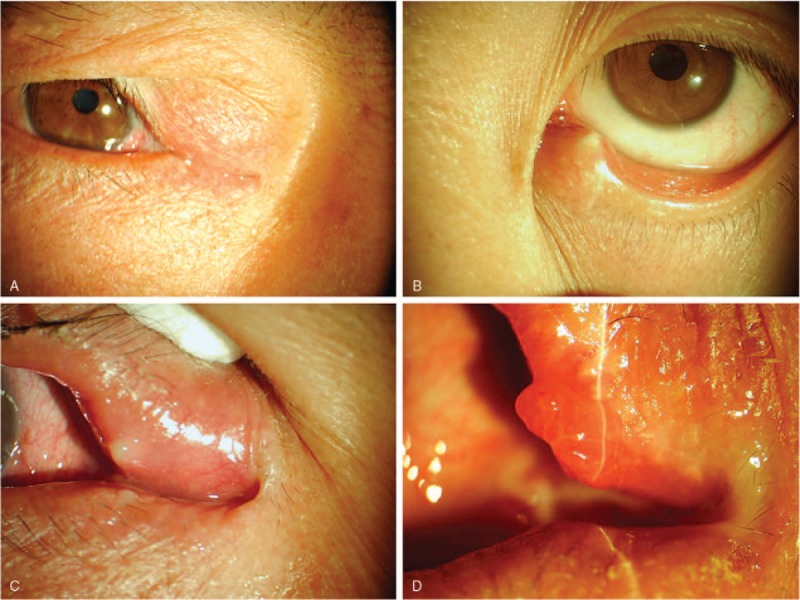
Clinical signs of canaliculitis concretions. (A) Medial canthal swelling of the affected eyelid. (B) Punctal dilation and mucopurulent discharge. (C) The upper punctum erythema and punctal regurgitation with mucopurulent discharge. (D) The affected eyelid inflammatory congestion and punctal granulomatous lesion.

Among the 37 consecutive primary canaliculitis patients enrolled, 1 was excluded for no concretions, and no secondary canaliculitis, so 36 (97.3%) patients had concretions and were eligible in the total (Table 2, Supplemental Digital Content 1). There were 30 (83.3%) female and 6 (16.7%) male patients with a mean age of 54.2 years (median 50 years; range, 30–95 years). Three patients had a history of dacryocystitis and were treated with dacryocystorhinostomy. All patients were presented with unilateral eye involvement. The left eye was involved in 21 patients (58.3%), and the right was involved in 15 patients (41.7%). Concretions were more likely to be found in the lower lids (23/36; 63.9% of cases), followed by upper canaliculus (10 cases), with both upper and lower involvement in 3 patients. Twenty-eight (77.8%) patients were misdiagnosed or delayed diagnosed; the mean duration of symptoms until diagnosis among the group with canalicular concretions was 17.1 months (median 8 months; range, 1–84 months). The main clinical manifestations were discharge (36cases, 100%), followed by epiphora (24 cases, 66.7%), erythema (19 cases, 52.8%), swelling (17 cases, 47.2%), irritation (13 cases, 36.1%), and pouting punctum (12 cases, 33.3%). Concretions or discharge from the punctum were found in 30 of 36 patients (83.3%) by massaging the medial aspect of the affected eyelid. Lacrimal syringing was patent in all cases, but irrigation of the affected canaliculus in 21 of 36 patients (58.3%) revealed some degree of resistance (Fig. 1).

**Table 2:**
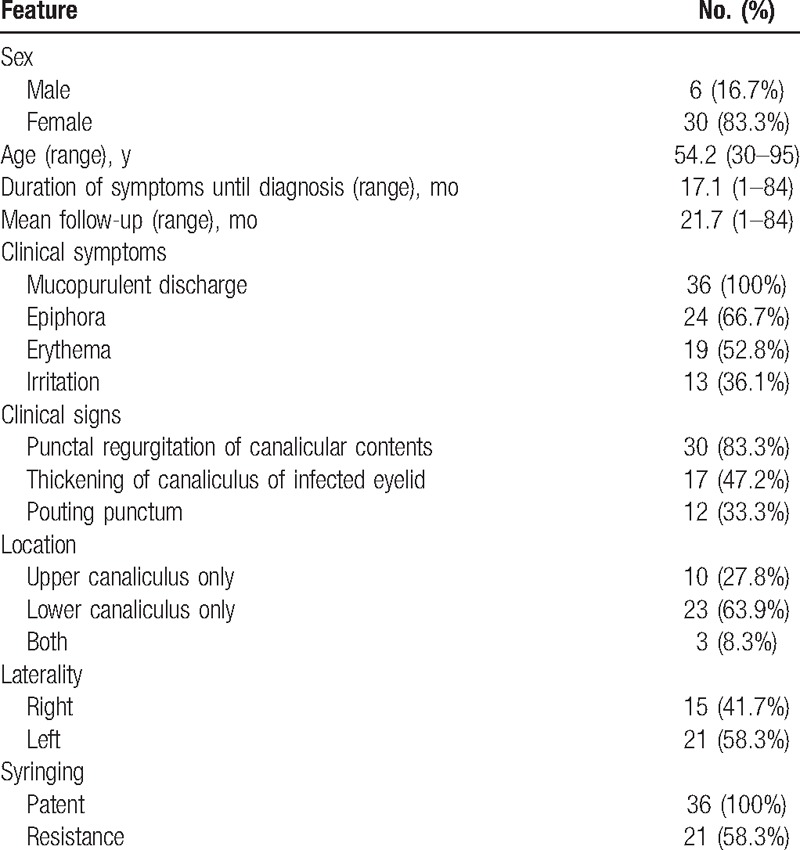
Clinical features in 36 patients of canaliculitis with concretions.

**Figure 2:**
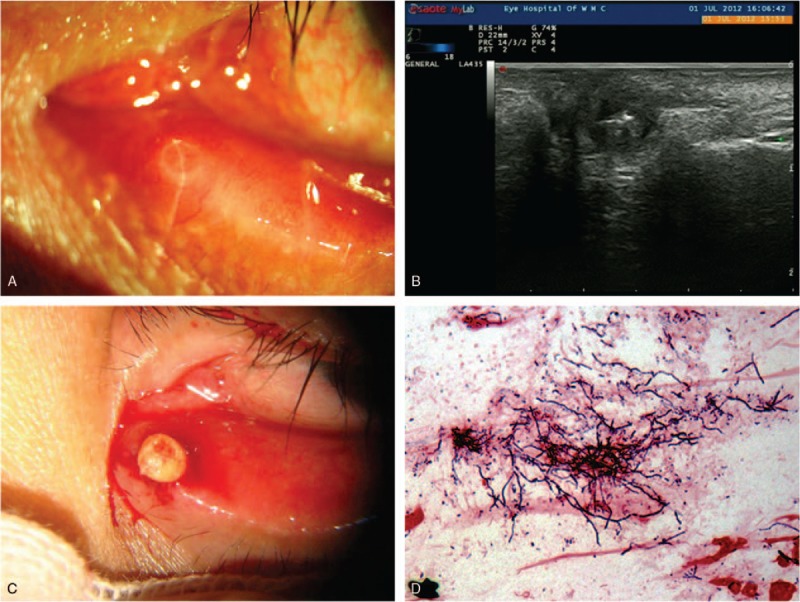
Clinical feature of canalicular concretions. (A) A swollen pouting punctum and eyelid erythema. (B) Ultrasonic images showed ectasia of the canaliculus and sulfur grains with a strong echo. (C) Incising the upper punctum and extruding concretions by retrograde expression. (D) The smear of concretion showed filamentous bacteria and then identified *Actinomyces* by microbiologic culture.

**Figure 3:**
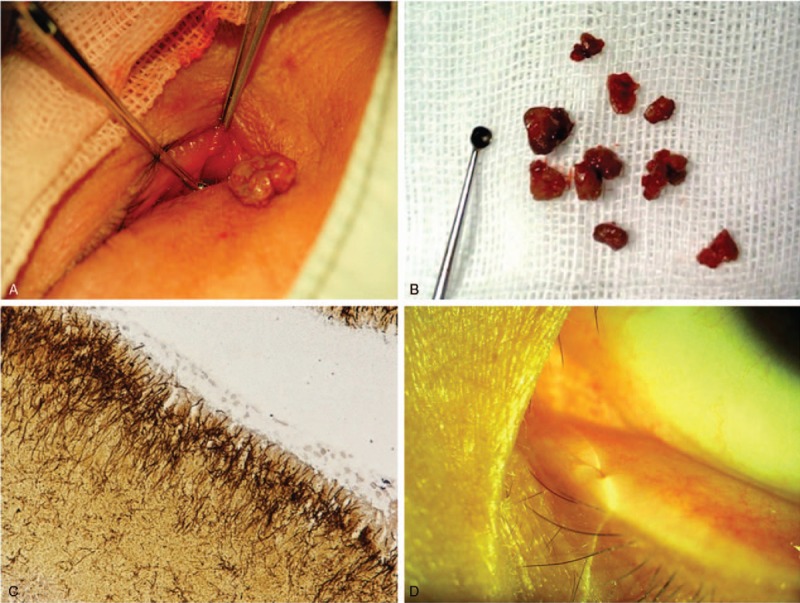
Surgery treatment and outcomes for canalicular concretions. (A) Surgery with canaliculotomy showed concretions. (B) Curetted all of concretions and canaliculus contents. (C) Histopathologic examination with Gomori methenamine silver staining showed filamentous bacteria (*Actinomyces*). (D) Minute scar after 3 weeks of surgery, and complete resolution of the symptoms.

Ultrasonic imaging was used in the diagnosis of concretions in 6 patients who were not classical typical clinical presentations. One patient was excluded because concretions were not found. Five canaliculitis patients’ ultrasonic images showed ectasia of the canaliculus and sulfur grains with a strong echo, which indicate the existence of a concretion (Fig. 2). During surgical treatment, canalicular concretions were readily removed from the canaliculus in all 36 patients. Culture of concretions was performed in 24 of 36 patients (66.7%). Microbiologic results were positive in 13 of 24 cases (54.2%), with *Streptococcus* species occurring in 4 patients (30.8%), *Staphylococcus* species occurring in 3 patients (23.1%), *Actinomyces* in 3 cases (23.1%), as well as *Aerococcus urinae*, *Citrobacter koseri*, and *Bacillus mirabilis* occurring in 1 patient each. Histopathologic examination was obtained in 13 patients and revealed *Actinomyces* species in 8 patients (Fig. 3). In the rest of the patients, only inflamed granulation tissues were revealed.

The average interval between surgery and telephonic follow-up was 21.7 months (range, 1–84 months). Among the telephonic questionnaires, 35 of 36 patients’ symptoms improved, with 33 patients having symptom resolution, 2 patients having decreased symptoms of epiphora for which they desired no further treatment; 1 patient developed a recurrence of canaliculitis after 10 months, and that patient experienced completed resolution after surgery with curettage and canaliculotomy. It was noted that these 3 patients had preexisting lacrimal pathology. None of these patients were noted to have developed canalicular scarring or narrowing subsequent to canaliculotomy.

## 4 Discussion

Dacryolithiasis (lacrimal sac dacryoliths or canalicular concretions), which is an often overlooked but important phenomenon, can occur in any part of the lacrimal system. Dacryoliths have been reported in between 6% and 18% of dacryocystitis cases.^[2,6,12–15]^ Ellis et al thought that the finding of concretions was diagnostic, and Pavilack and Frueh^[3]^ established the diagnosis of chronic canaliculitis by the presence of expressible canalicular concretions. Lee et al also thought that concretions extruding from the punctum were an important characteristic and a sign for diagnosis with canaliculitis.^[16,17]^In addition, a series of case studies summarized the higher incidence rate between 73.3% and 100% of canalicular concretions in canaliculitis patients.^[2–8,17]^ In our current study, concretions were identified in 36 of 37 cases (97.3%), and most of the patients had >1 piece of concretion, mainly containing approximately 4 to 8 pieces. However, few studies noted a lower rate, but the authors did not explain the reasons for the low incidence.^[10,11]^ Overall, however, the proportion of concretions in canaliculitis patients is still higher, so canalicular concretions is very common in primary canaliculitis.

The clinical features of primary canaliculitis with concretions in our cases are somewhat similar to the published reports in Table 1, with the main difference being the incidence of symptoms, including a greater number of female patients, mean patient age, side and location of eyelid, and duration of symptoms until diagnosis.^[4,18,19]^ In our current study, the clinical symptoms are discharge in angulus oculi medialis in the morning (100%), followed by epiphora (24 cases, 66.7%), erythema (19 cases, 52.8%), swelling (17 cases, 47.2%), irritation (13 cases, 36.1%), pouting punctum (12 cases, 33.3%), and concretions or discharge extruding from punctum (30 cases, 83.3%). Perumal and Meyer reported the main symptoms being swelling and erythema; Pavilack and Frueh and Repp et al reported the main symptoms being epiphora, discharge, swelling, and unilateral conjunctivitis.^[3,6,7]^ Hussain et al reported the main symptoms being chronic irritation, discharge, epiphora; Briscoe et al reported the main symptoms being epiphora, chronic conjunctivitis, swelling, and discharge.^[4,5]^ However, all of the previously mentioned findings were based on a small sample of cases.

Although the clinical spectrum of canaliculitis with concretions is well documented, there is still a high rate of misdiagnosis and delayed diagnosis. The average duration of symptoms until diagnosis was 17.1 months (1–84 months) in our study and ranged from 6 to 36 months in other published reports, reflecting the difficulty in diagnosis for clinicians.^[5,10,20]^ The apparent difficulty in diagnosis may be attributed to relative rarity of canaliculitis, which accounts for only 2% to 4% of all patients with lacrimal disease.^[2,9]^ Furthermore, canaliculitis may present without the classical clinical features, leading to additional difficulties in diagnosis. To reduce the misdiagnosis, we should know the clinical features of canaliculitis with concretions, but we should also recognize that concretions usually discharge by extruding from the punctum by pressing of the involved canaliculus and not of lacrimal sac compression.^[3,8]^

Sometimes we can use ultrasonic to get further information if we are not sure about the existence of concretions. High-resolution ultrasonic examination of the lacrimal drainage system demonstrated that a 20-MHz scanner is able to show ectasia of the canaliculus or sulfur grains measuring 1 to 2 mm in diameter (abnormal resoundings in canalicular), which indicated the existence of concretion. In 5 patients who did not display typical clinical presentations, ultrasonic examination showed sulfur grains in canaliculus, and the concretions were subsequently identified at surgery. Therefore, ultrasonic can offer a noninvasive means of identifying concretions and may improve the diagnostic accuracy.^[21,22]^

*Actinomyces* were thought to be the most common infectious agents.^[1,3–5,20]^ However, it was not cultured as frequently as we would have expected in our cases; only 3 of 13 cases were in culture-positive patients, so it may be attributed to the culture medium and methods.^[4,5]^ Histopathological examination is the most commonly reported diagnostic test for finding *Actinomyces*, as seen in 8 of the 13 specimens in our study.^[5,9,23]^ In addition, others have postulated that metabolic factors such as high calcium and phosphate levels within an obstructed lacrimal system may contribute to their formation.^[13]^ However, the study has not been conducted with a formal statistical comparison between concretion and nonconcretion groups for microbiological profile or other clinical variables.^[11]^ Our series also does not find any significant risk factors to be associated with the presence of concretions except age and female.

Conservative therapy such as topical and/or systemics antibiotics, digital massage, corticosteroids, irrigation, syringing, and even curettage by punctum alone, was reported to have a high probability of recurrence among canaliculitis with concretions patients.^[1,3,8,10,11]^ Lin et al^[11]^ confirmed that concretions were noted in all patients with previous concretions during a recurrent attack. The concretions may prevent antibiotics from eradicating the bacterial source by virtue of obstruction of the flow and protection of bacteria within the stones, which serve as a reservoir for bacteria; thus, it is an important risk factor for the development of recurrent or persistent canaliculitis. Therefore, more aggressive treatments such as canalicular curettage after canaliculotomy or punctoplasty are recommended to completely remove all concretions, which may be necessary and beneficial to reduce recurrence. In cases with concretions, the surgical removal of all possible concretions is essential for cure.

In this study, all of our cases underwent surgery with curettage after canaliculotomy to completely remove all concretions and contents in the canaliculus. All patients’ symptoms improved, with 33 of 36 patients having symptom resolution, 2 patients having decreased symptoms of epiphora, and 1 patient having recurrence. Canaliculotomy is favored for excellent exposure, greater access, and easier curettage of canalicular contents, and it is highly efficacious in treating canaliculitis, with complete resolution occurring in the majority of primary canaliculitis patients.^[1,5,10,11,23]^ Risks of canaliculotomy include canalicular luminal narrowing or scarring, lacrimal pump dysfunction, and canalicular fistula formation, but none of our patients were noted to have developed canalicular scarring or narrowing and lacrimal pump dysfunction at their 3-week follow-up in our series.^[2,24]^ In addition, the recurrence of canaliculitis with concretions after canaliculotomy was reported in a previous study^[8,11]^; however, there was only 1 patient's recurrence of the symptoms at median 21.7 months’ follow-up according to the telephonic questionnaires. An experienced and skillful surgeon may also decrease the risks of operation complication and recurrence. Therefore, many experts suggested that curettage and canaliculotomy are the standard therapy for canaliculitis with concretions, and our study also confirms this view with a large sample.^[8,11,23]^

In summary, we should recognize the classic clinical features of concretions, extrude the involved canaliculus, which can help to better detect the concretions. Sometimes, we can use ultrasonic to verify the existence of concretions. Microbiologic culture and histologic examination can find *Actinomyces* or other bacteria species. Canaliculotomy with curettage is the standard therapy for concretions with canaliculitis, and surgical removal of all possible concretions is essential for cure.

## Supplementary Material

Supplemental Digital Content
