# Supplementary material for: Clinical features and surgical outcomes of primary canaliculitis with concretions
Source: Medicine (Baltimore). 2017 Mar 3;96(9):e6188. doi: 10.1097/MD.0000000000006188 (PMC5340447; doi:10.1097/MD.0000000000006188)

Supplemental Digital Content 1. Clinical pictures of 36 canalicular concretions patients.

All case images of Canalicular Concretions

Case 1 Female 79yrs Date of surgery：1. 31. 2008

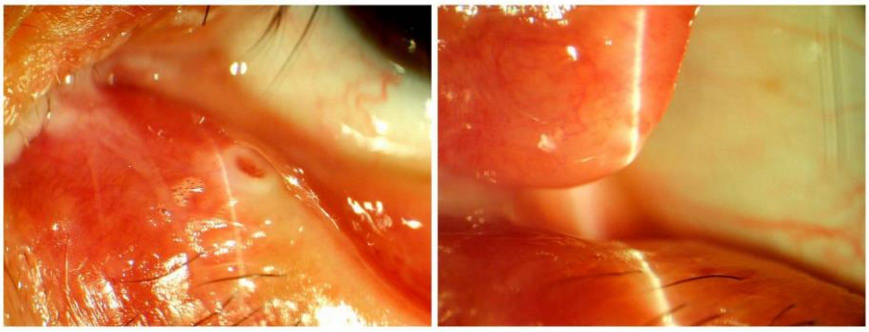

Case 2 Female 61yrs Date of surgery：10. 21. 2008

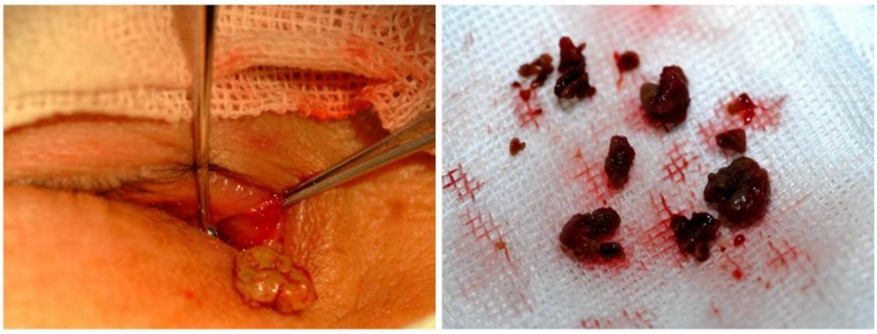

Case 3 Female 41yrs Date of surgery：4. 9. 2009

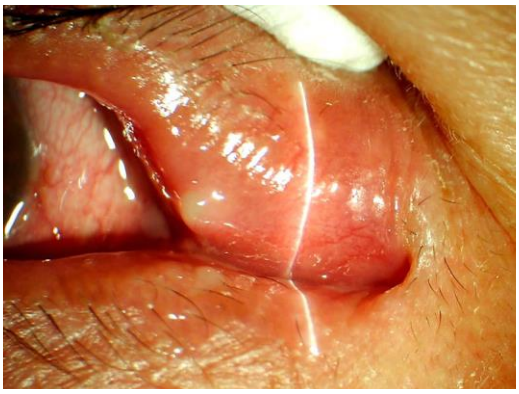

Case 4 Male 54yrs Date of surgery：6. 18. 2009

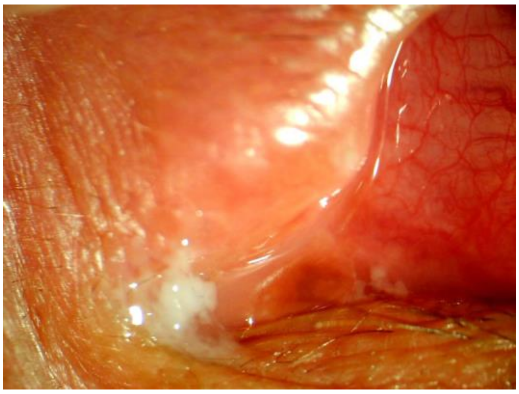

Case 5 Male 35yrs Date of surgery：9. 21. 2010

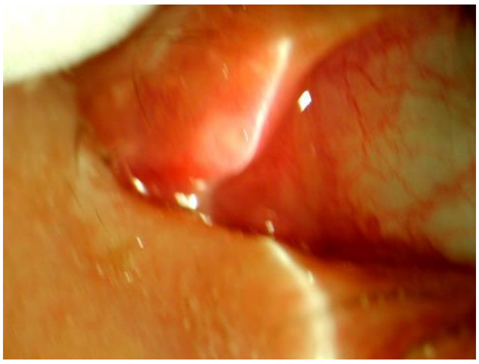

Case 6 Female 32yrs Date of surgery：10.10. 2010

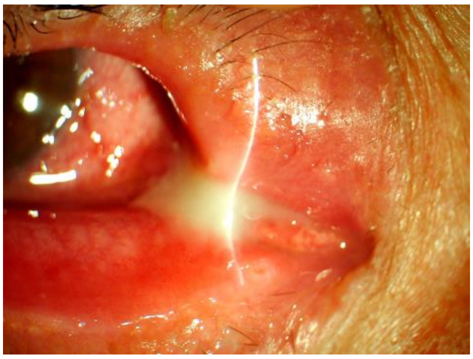

Case 7 Female 46yrs Date of surgery：1. 24. 2011

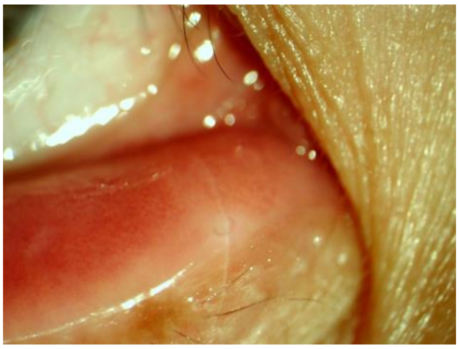

Case 8 Female 76yrs Date of surgery：2. 14. 2011

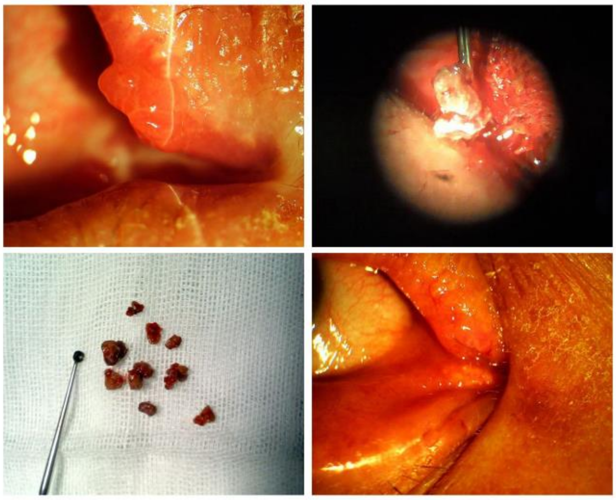

Case 9　　Female　　85yrs　　Date of surgery：3. 17. 2011

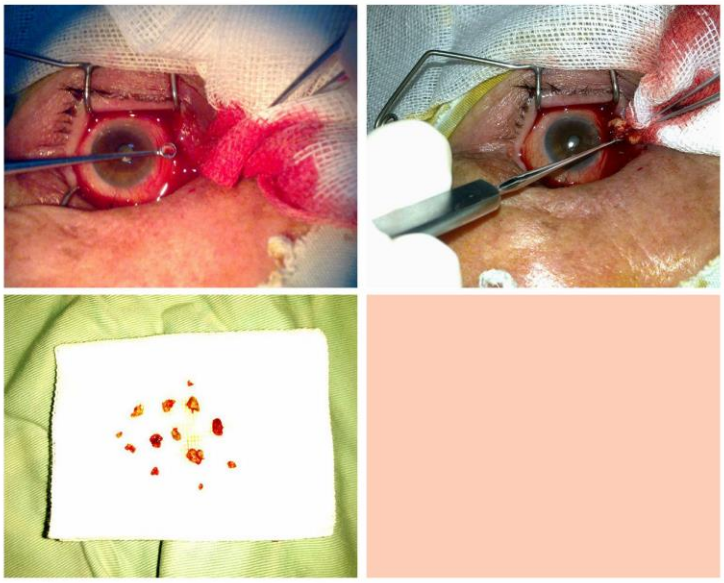

Case 10　　Female　49yrs　Date of surgery：6. 19.　2011

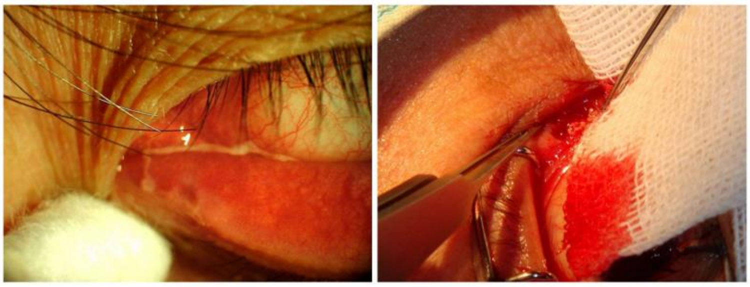

Case 11　　Male　82yrs　　Date of surgery：8. 25. 2011

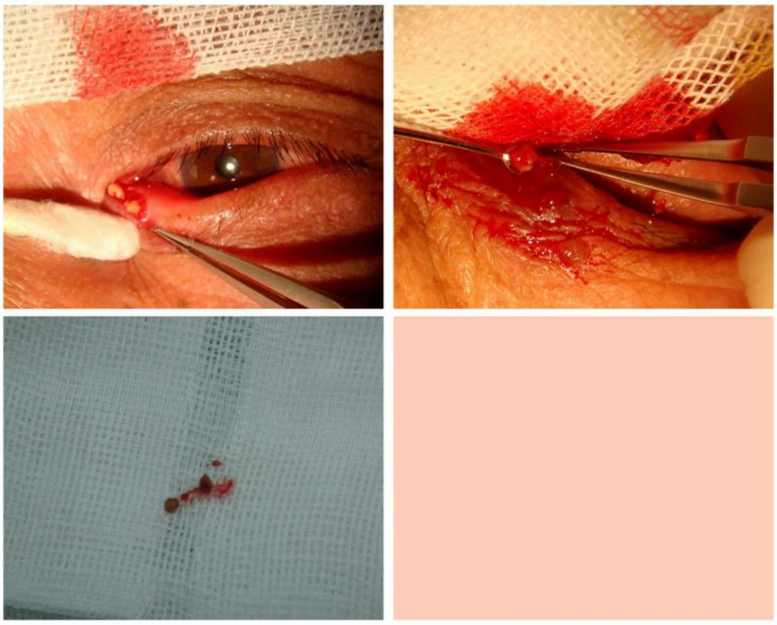

Case 12 Female 78yrs Date of surgery：9.4.2011

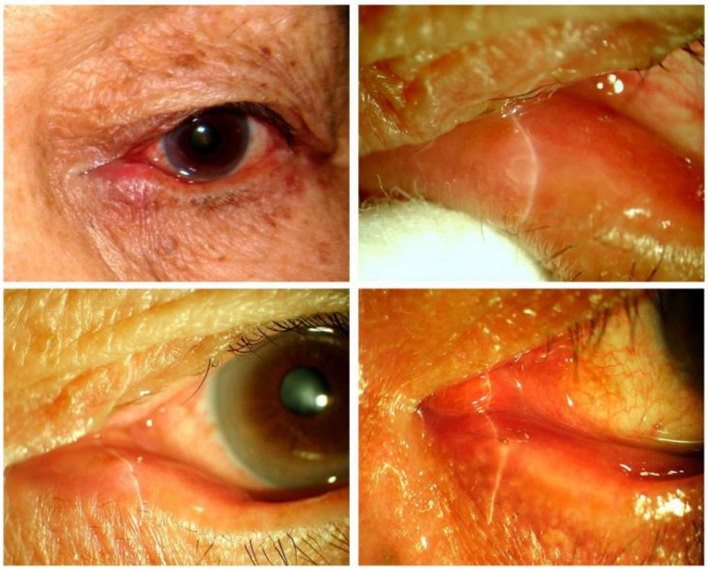

Case 13 Female 78yrs Date of surgery：11. 22. 2011

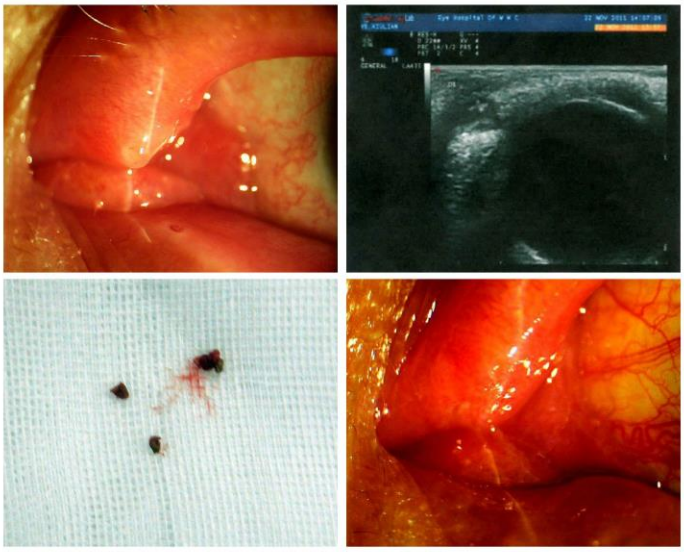

Case 14 Female 59yrs Date of surgery：11. 22. 2011

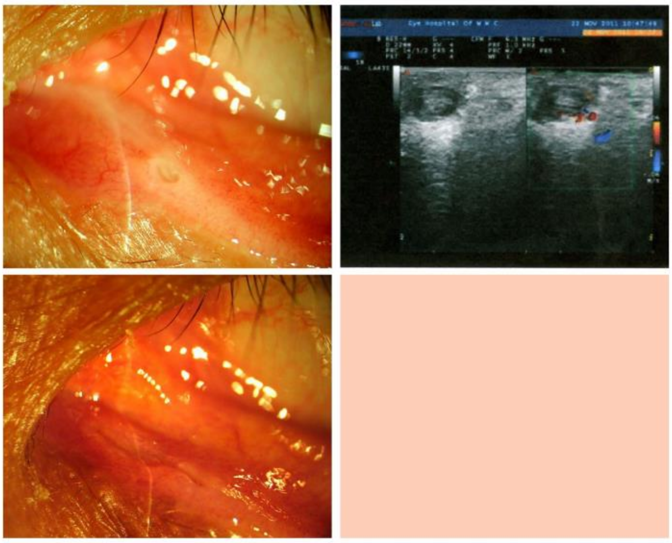

Case 15 Female 53yrs Date of surgery： 12. 5. 2011

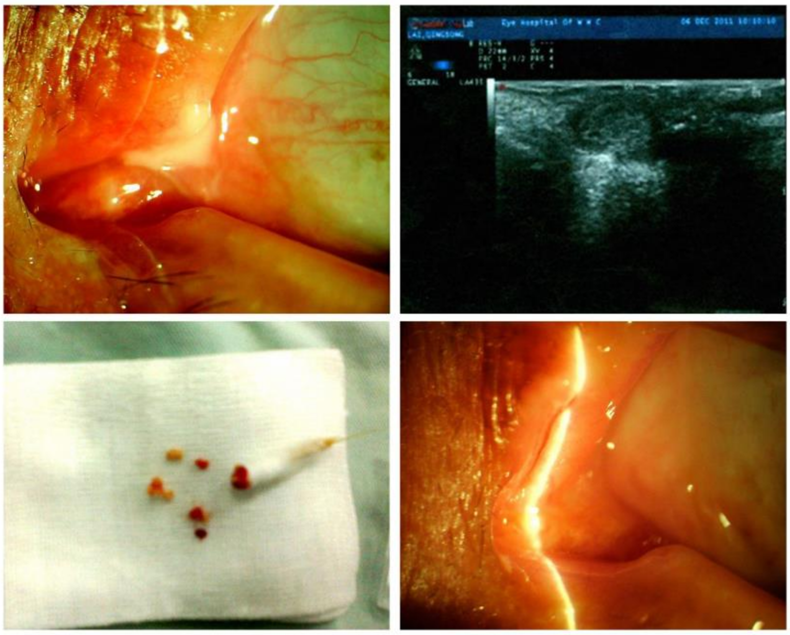

Case 16 Female 56yrs Date of surgery：12. 20. 2011

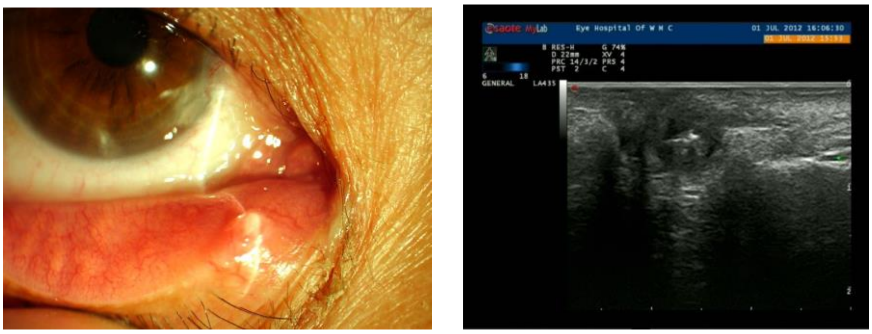

Case 17 Male 39yrs Date of surgery：3. 12. 2012

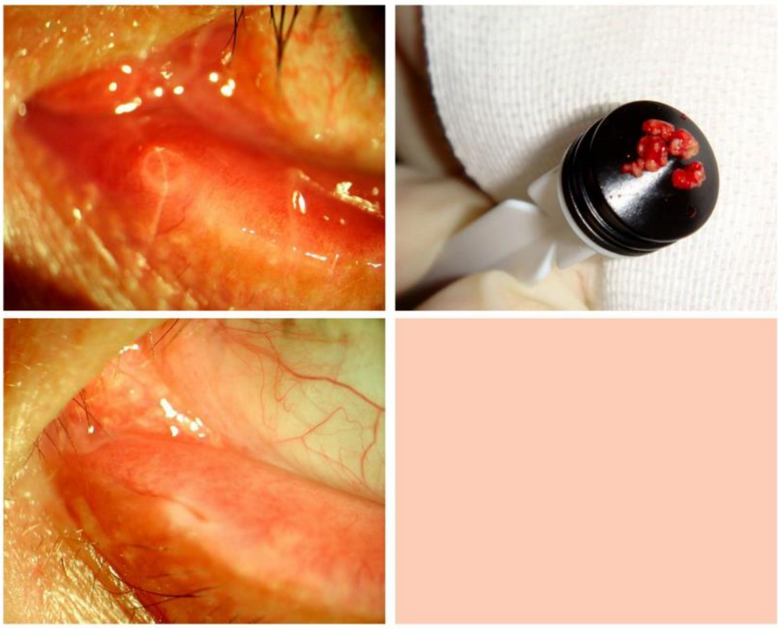

Case 18 Female 30yrs Date of surgery：6. 28. 2012

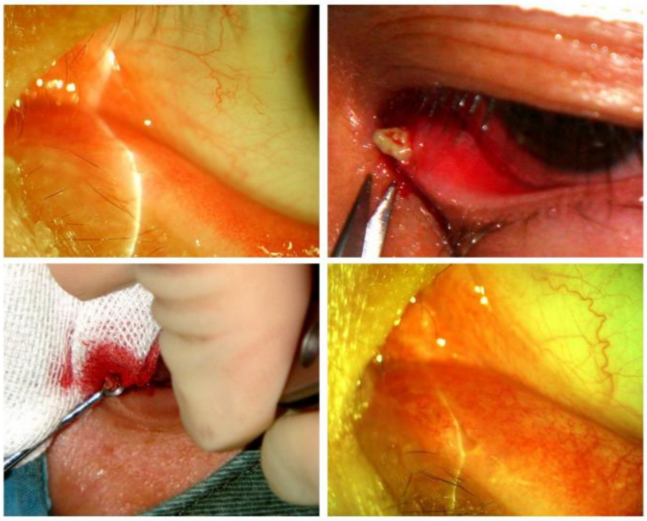

Case 19 Female 38yrs Date of surgery： 9. 2. 2012

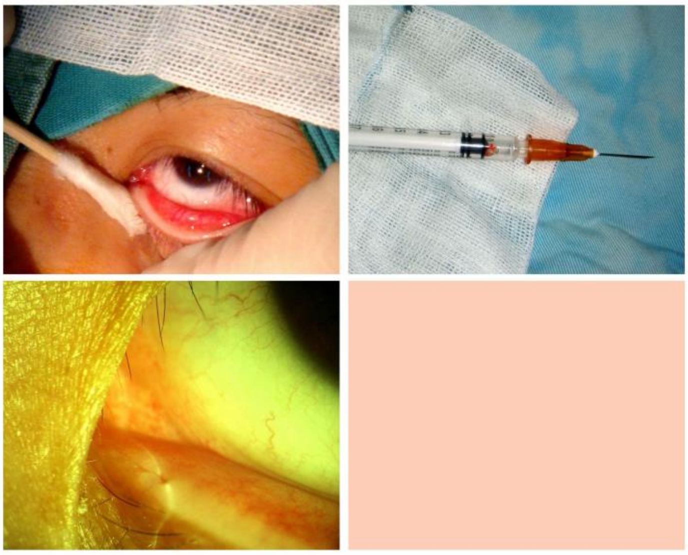

Case 20 Female 51yrs Date of surgery：11. 15. 2012

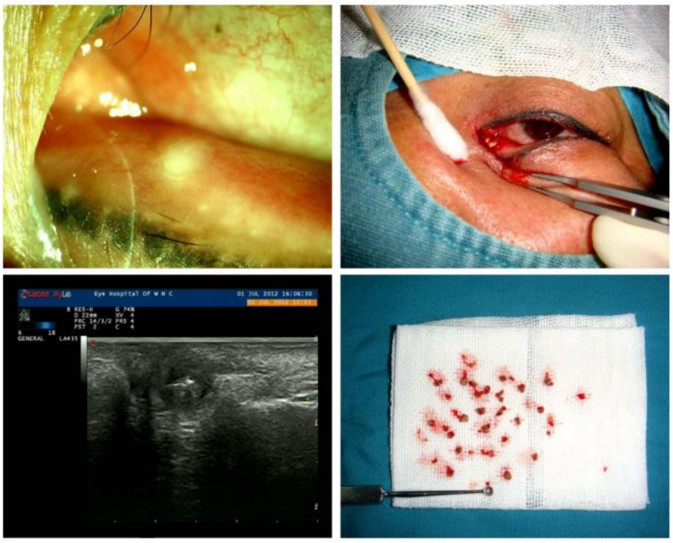

Case 21   Female   77yrs   Date of surgery：12. 6. 2012

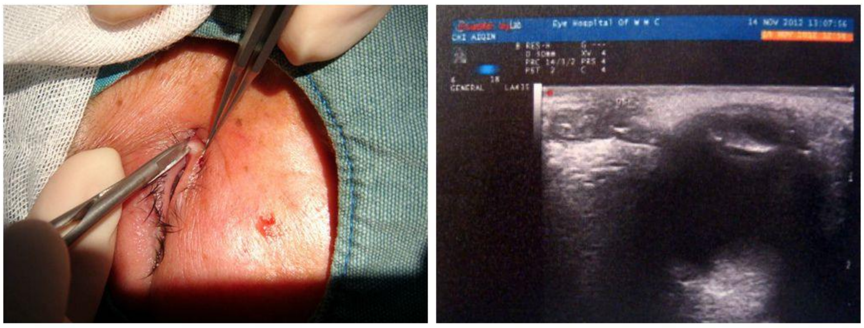

Case 22   Female   47yrs   Date of surgery：5. 30. 2014

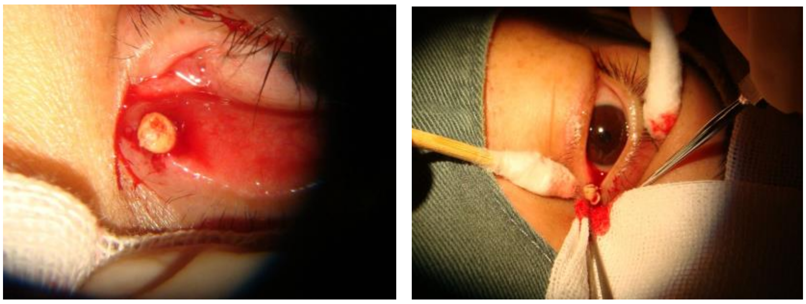

Case 23   Female   42yrs   Date of surgery：6. 7. 2014

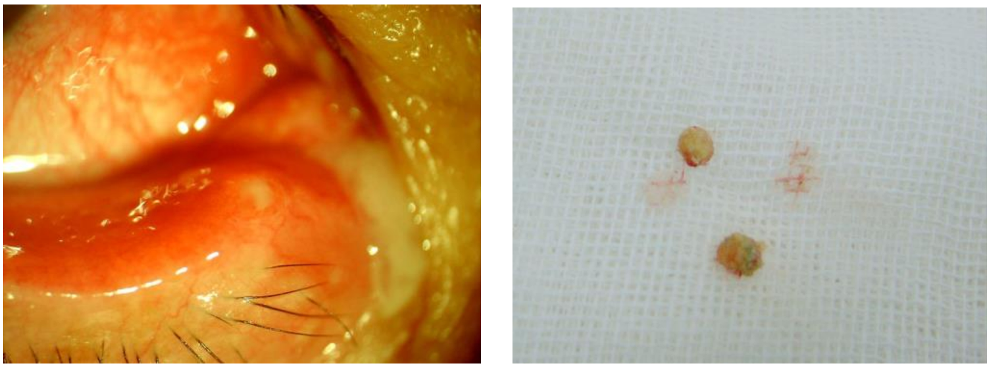

Case 24   Female   52yrs   Date of surgery：9. 1. 2014

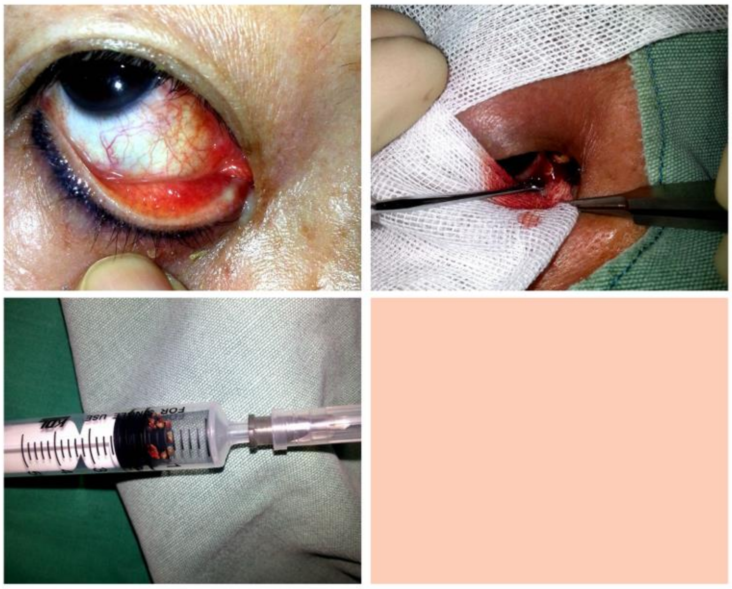

Case 25 Female 34yrs Date of surgery：10. 3. 2014

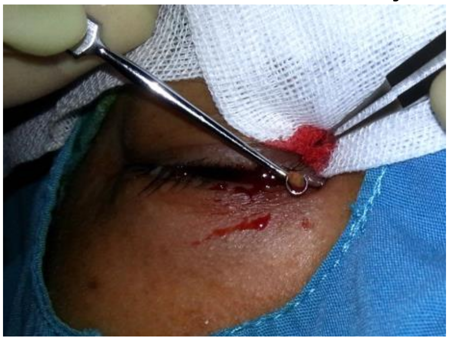

Case 26 Female 44yrs Date of surgery：10.7 .2014

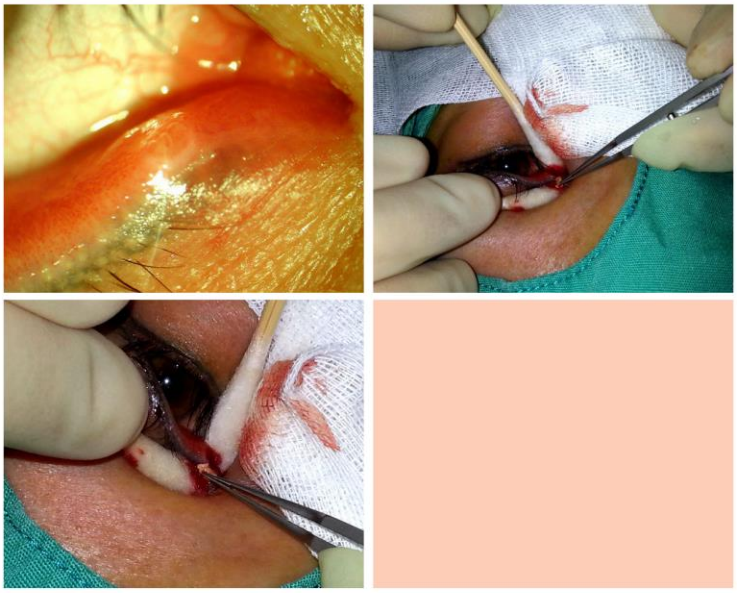

Case 27 Male 30y Date of surgery：11. 25. 2014

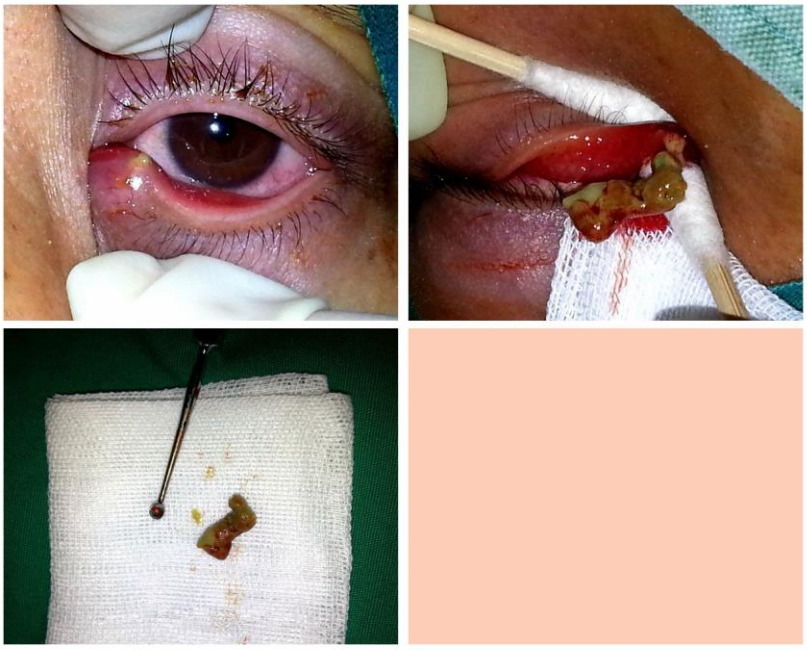

Case 28 Male 81yrs Date of surgery：5. 30. 2015

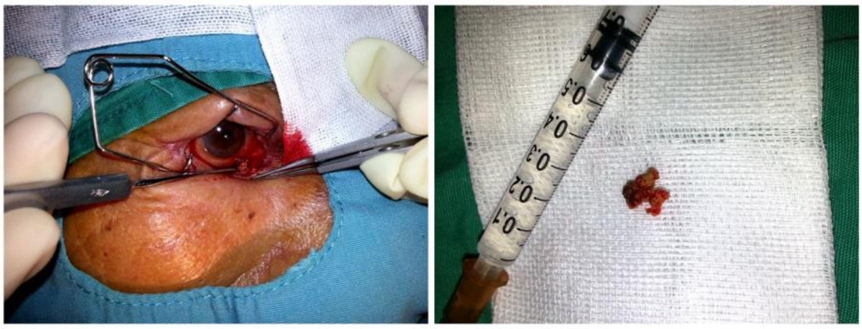

Case 29 Female 50yrs Date of surgery：7. 3. 2015

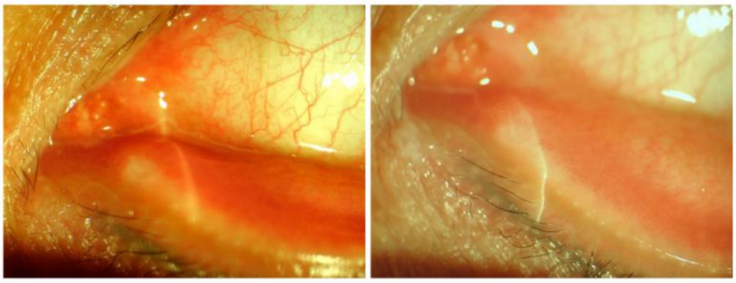

Case 29 Female 62yrs Date of surgery：7. 16. 2015

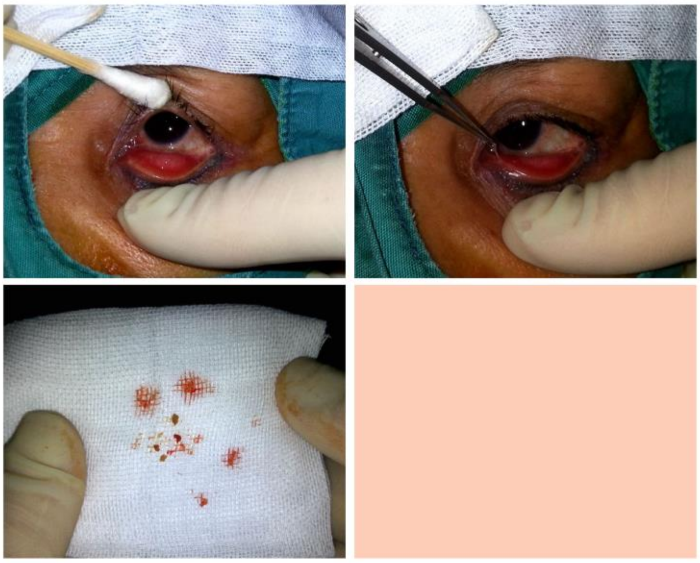

Case 31 Female 76yrs Date of surgery：11. 17. 2015

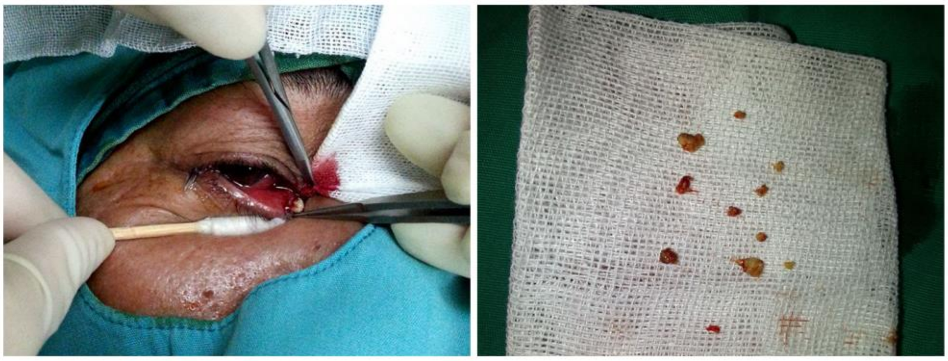

Case 32 Female 95yrs Date of surgery： 2. 22. 2016

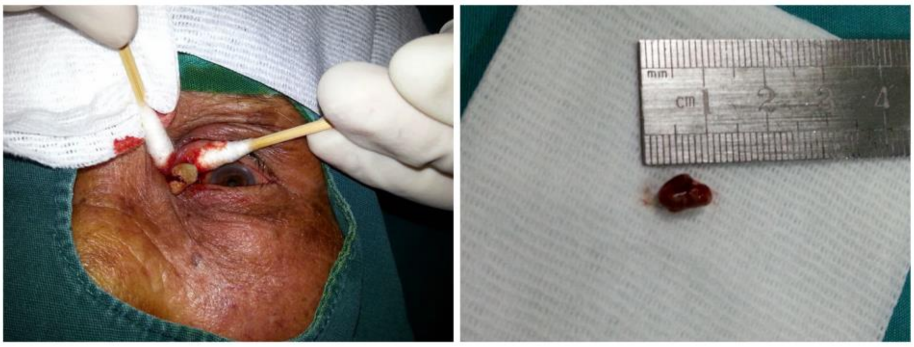

Case 33 Female 40yrs Date of surgery：4. 18. 2016

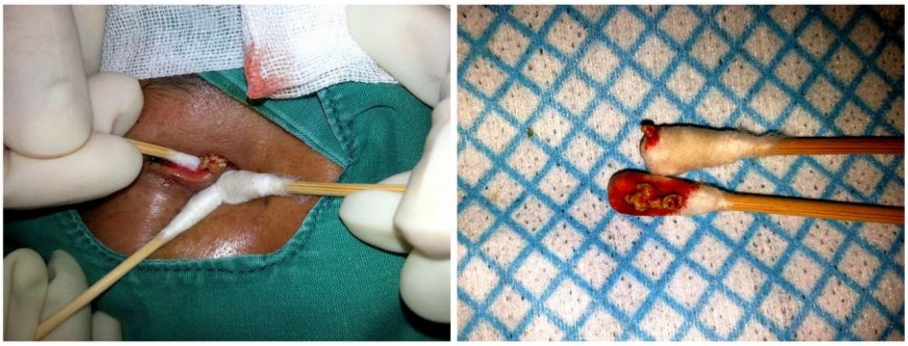

Case 34 Female 47yrs Date of surgery： 6. 7. 2016

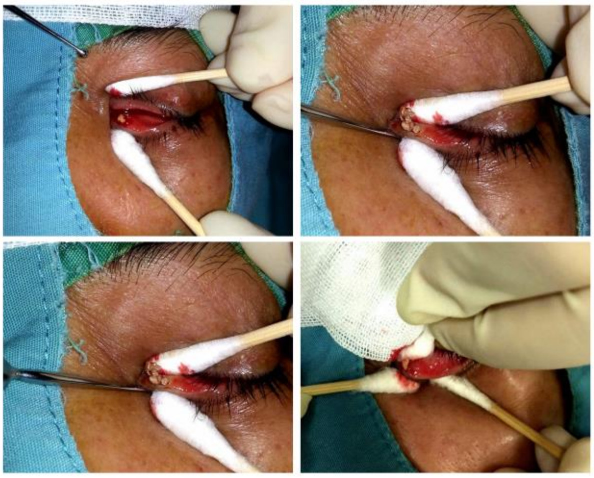

Case 35 Female 42yrs Date of surgery：6. 23. 2016

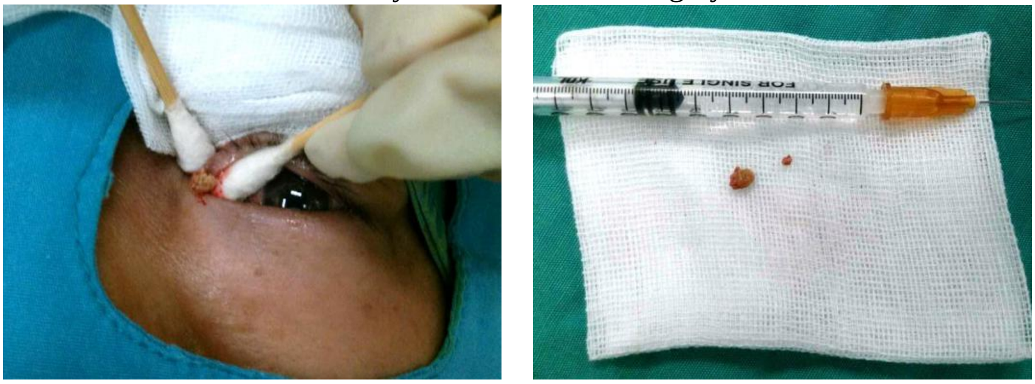

Case 36 Female 49yrs Date of surgery：7. 21. 2016

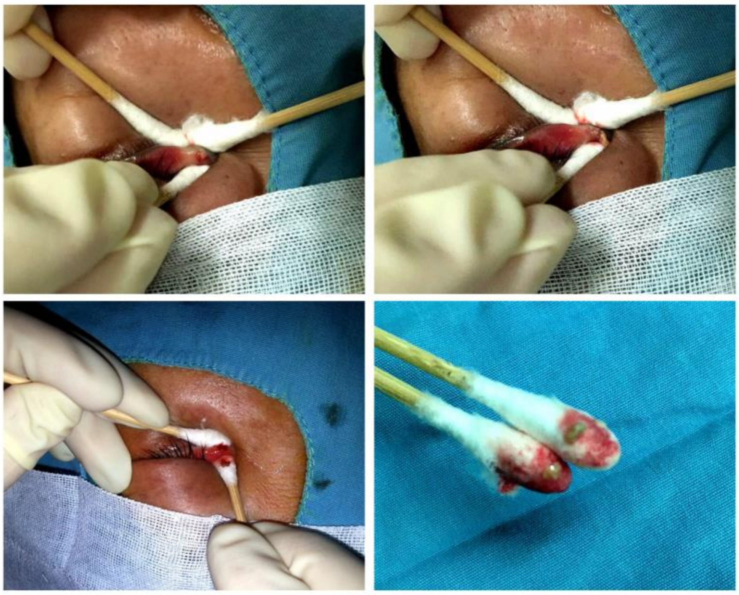

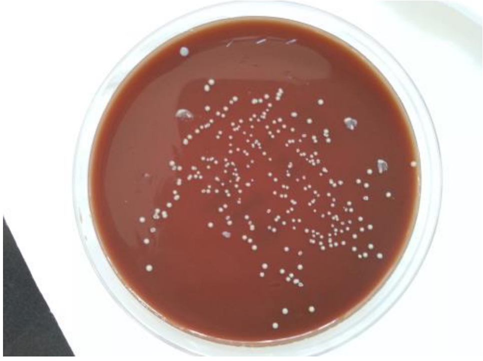

Case 37 Famale 37yrs Date of surgery：8.12.2016

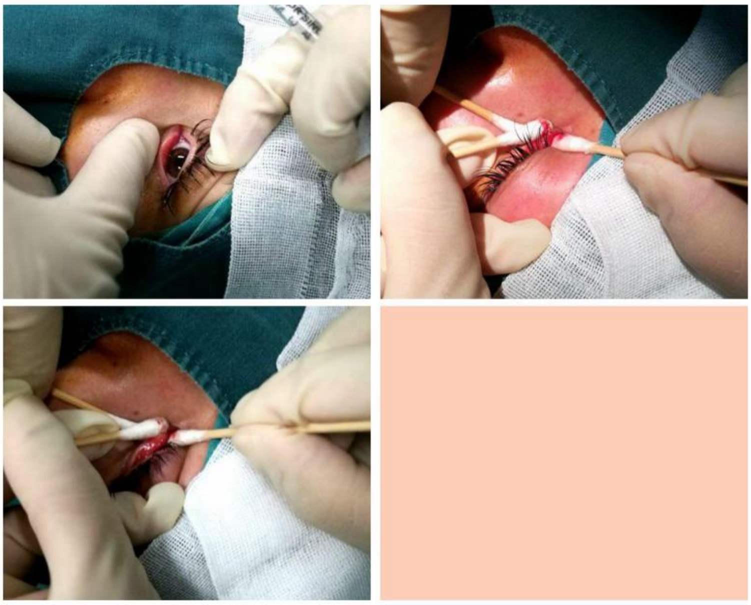

Supplement: Supplemental Digital Content [file medi-96-e6188-s001.pdf]
